# Inkjet‐Printed Physical Unclonable Functions For Secure Authentication

**DOI:** 10.1002/smll.202514908

**Published:** 2026-04-30

**Authors:** Riccardo Sargeni, Elisabetta Dimaggio, Francesco Pieri, Stefano Di Pascoli, Giuseppe Iannaccone, Gianluca Fiori

**Affiliations:** ^1^ Dipartimento di Ingegneria dell' Informazione Universitädi Pisa Pisa Italy; ^2^ Quantavis s.r.l. Largo Padre Renzo Spadoni Pisa Italy

**Keywords:** counterfeiting, inkjet printing, physical unclonable functions

## Abstract

Counterfeiting is a growing global challenge with significant economic and social implications. Physical Unclonable Functions (PUFs), exploiting manufacturing randomness to generate unique and unclonable identifiers, have emerged as a promising solution for secure authentication. This study presents a novel, scalable method for fabricating inkjet‐printed PUFs by exploiting the randomness of ink droplet deposition on substrates such as paper. By optimizing geometric features, the proposed system ensures high uniqueness, reliability, and bit uniformity. The PUFs also exhibits excellent durability, maintaining performance under mechanical stress and chemical exposure. Furthermore, the system incorporates a low‐cost imaging setup and advanced positional markers, enabling fast and accurate database validation. This work establishes a robust and low‐cost route to PUFs that can be interrogated with consumer‐grade devices, making them suitable for various anticounterfeiting applications, including supply chain security and luxury goods authentication.

## Graphical abstract

Herein, we propose a low‐cost authentication system based on a scalable fabrication technology and user friendly recognition system. It involves inkjet printing as a manufacturing method and an optical recognition system based on contour features extraction. An encapsulation method is proposed to give mechanical and chemical stability to the tag.

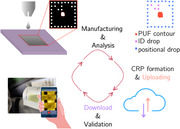

## 1 Introduction

Counterfeiting is a growing global challenge, with an estimated annual trade of counterfeit goods of 3.3% of the global trade (450 billion EUR [1]), similar in size to the total revenues of the semiconductor industry, which is approximately 600 billion EUR [2]. In the European Union, counterfeit and pirated products account for about 5% of all imports, resulting in serious economic consequences, lost revenues, and threats to consumer safety [1]. This economic impact highlights the urgency for innovative measures, including the use of physical properties of materials to combat counterfeiting [3].

Physical Unclonable Functions (PUFs) are among the most promising solutions for secure authentication, using the inherent randomness of manufacturing processes to encode unique, unclonable keys [4, 5, 6, 7, 8, 9, 10]. This randomness provides high security by making cloning virtually impossible, while the unique physical characteristics of each device significantly reduce the likelihood of duplication.

Commercial PUFs are typically based on intellectual property that can be instantiated in integrated circuits [11, 12] and are used as a hardware Root of Trust, provisioning and storing cryptographic root keys for authentication.

However, in many real‐life situations, it would be very useful for normal citizens to verify the authenticity of goods and documents in their life using consumer‐grade devices, such as smartphone cameras with or without simple add‐ons. In addition, the use of physical authentication tags, relying on non‐electronic and non‐digital properties, would make the PUF solution inherently more robust to AI‐based attacks that in the world of bits try to reproduce the algorithms [13].

Advances in PUF technology over the past decade, particularly in optical [14, 15, 16, 17, 18, 19, 20] and hybrid PUFs using photonic crystals and plasmonic metamaterials, have expanded the landscape of secure authentication [21]. Thus, there is a critical need for high‐entropy, low‐cost, and robust PUFs that can be scaled for practical applications.

Nanomaterials have shown considerable potential as unique markers in PUFs [22]. Approaches using quantum dots [23, 24], Raman fingerprints of nanoparticles [25], and grain boundaries in 2D materials [26] offer innovative ways to address security challenges. Memristors [27], monolayer ${\mathrm{MoS}}_{2}$ transistors based on memristors [28] and inkjet printed PUFs that employ transistor variability [28, 29, 30, 31] have also emerged as promising directions. Alternative methods, such as random fractal PUFs on lithographically fabricated gold films [32] or structural color inkjet‐printed PUFs with AI‐based authentication [33, 34, 35, 36], while innovative, are limited by higher cost and manufacturing complexity. These approaches exploit random physical variations to create secure, device‐specific identifiers for authentication.

Biological PUFs, due to the randomness of cell growth processes, demonstrate unique and low reproducibility authentication tags [37]. Nanoparticle‐based ink has been employed to create fingerprint patterns via random drop‐casting methods [38] or to create a unique resistivity network [39, 40]. Fractal PUFs based on lithographically fabricated thin films offer tunable complexity, but are limited in their adaptability to flexible substrates [32].

In this study, we propose a scalable, efficient, and cost‐effective method to fabricate PUFs using the randomness generated during inkjet printing. To the best of our knowledge, our work is the first to exploit the contour of inkjet printed features as micro‐scaled fingerprint. The shape of the ink droplet is influenced by its rheological properties, the surface roughness of the substrate, and drying dynamics, creating patterns that are both highly random and inherently unique. These patterns, typically tens to hundreds of microns in size, can be imaged using simple tools, such as a microscope or a smartphone camera with a magnifying lens. The captured images are then compared to those stored in a cloud database for authentication. Unlike traditional methods, that rely on complex read‐out systems, [33, 36, 41], are limited to one class of application [39, 42] and not‐scalable authentication methods [18] and propose complex structures or material systems [32, 43], this approach is simple, scalable, accurate, and multi‐purpose [44, 45, 46]. The proposed method enables the deposition of ink droplets with sub‐micron precision, ensuring robust and reproducible results that are resistant to counterfeiting attempts. This randomness is consistent with efforts to exploit nanoscale variability for secure applications, such as defect‐driven randomness in 2D materials [26, 47]. To quantify the performance of the proposed PUFs, we analyze key metrics such as uniqueness, bit aliasing and uniformity, and relate these parameters to the underlying entropy of the generated patterns [13, 48, 49]. By using entropy‐based thresholding methods for contour detection [50, 51], we ensure high accuracy and robustness in the authentication process. This work establishes a scalable route to low‐cost, robust, and easily verifiable PUFs that are resistant to modern counterfeiting techniques, providing practical solutions for secure authentication in applications ranging from supply chain management to luxury goods authentication.

## 2 Results and Discussion

### 2.1 Fabrication of Inkjet Printed PUFs

**FIGURE 1:**
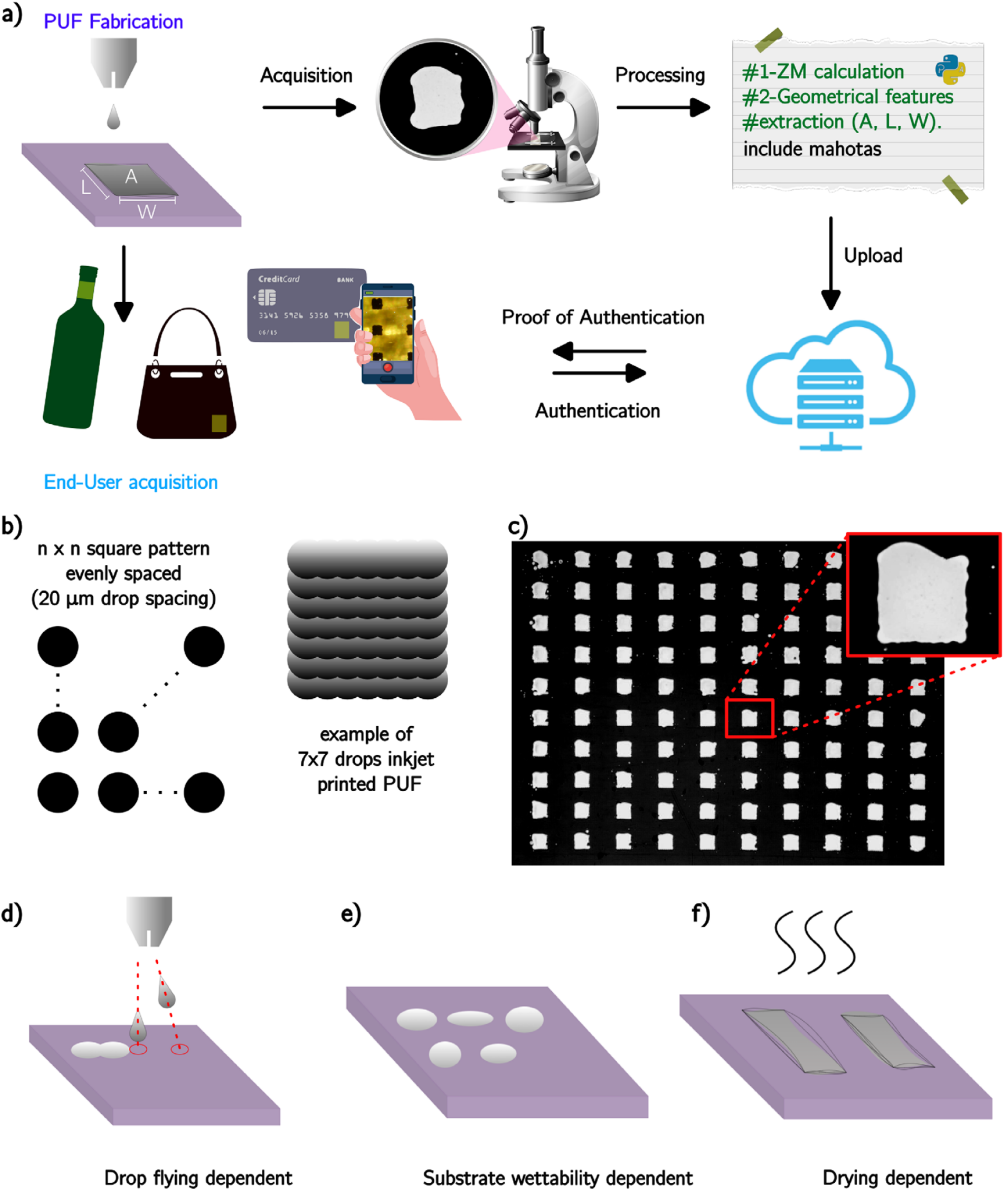
(a) PUF fabrication, acquisition, and analysis, with validation between manufacturer Zernike Moment (ZM) and end‐user acquired ZM; (b) a possible pattern for the printed PUF; (c) a matrix of printed PUFs with an enlargement on a single element; a list of uncertainties derived from the (d) drop falling from the nozzle onto the substrate, (e) the substrate wettability, (f) the drying process of the ink after printing.

The workflow of our inkjet printed PUFs is shown in Figure 1a. Starting with the deposition of an ultra‐thin polyimide film on a silicon wafer, square patterns representing the PUF with a nominal area of 100 × 100 $\mu m^{2}$ are printed and then acquired with a End Microscope (HEM): the images are captured directly in a greyscale Portable Network Graphics (PNG) format to save storage space, with a resolution of 1600x1200 px in a field of view of 400 × 300 μm 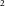, (further details can be found in the Section Acquisition Systems of the Supporting Information). For the processing of the acquired images, a calculation of the unique image moments is required. This was done with a Python script as in ref. [43]. The script uses Mahotas' Zernike Moment s (ZMs) function to extract the linearly independent ZM up to eight order associated with the contour of the PUF, for which pre‐processing steps such as blurring and OTSU's adaptive thresholding are necessary [52] in order to achieve a clear contour detection. The ZMs are stored in a safely‐shared database. The identification method allows the end‐user to acquire and validate the authenticity of the product, for example using a commercial smartphone zoom lens: from the low resolution image it is still possible to extract the ZMs and compare it with the stored ones, as it is shown in Section PUF characteristic parameters. Figure 1b shows an ideal print pattern for the PUFs, using 20 μm as the drop spacing, and an example of a 7 × 7 drop PUF sketch. Figure 1c shows a matrix of printed PUFs with an enlargement on a single element. The area of each element can be varied by setting the nominal printing parameters (drop spacing and number of drops), but the variability that ensures the uniqueness of each PUF is due to three main factors:

- the uncertainty of the point where the drop is deposited on the substrate, due to the distance between nozzle and substrate and the random trajectory of the drop, as shown in Figure 1d;
- the substrate wettability and the rheological properties of the ink, which eventually lead to uncertainty in the printed feature shape, as shown in Figure 1e;
- the speed of the drying process, which might distort the contours, as shown in Figure 1f.

These three random effects have a major impact on the shape of the PUFs, generating a huge number of possibilities.

### 2.2 PUF Configuration

**FIGURE 2:**
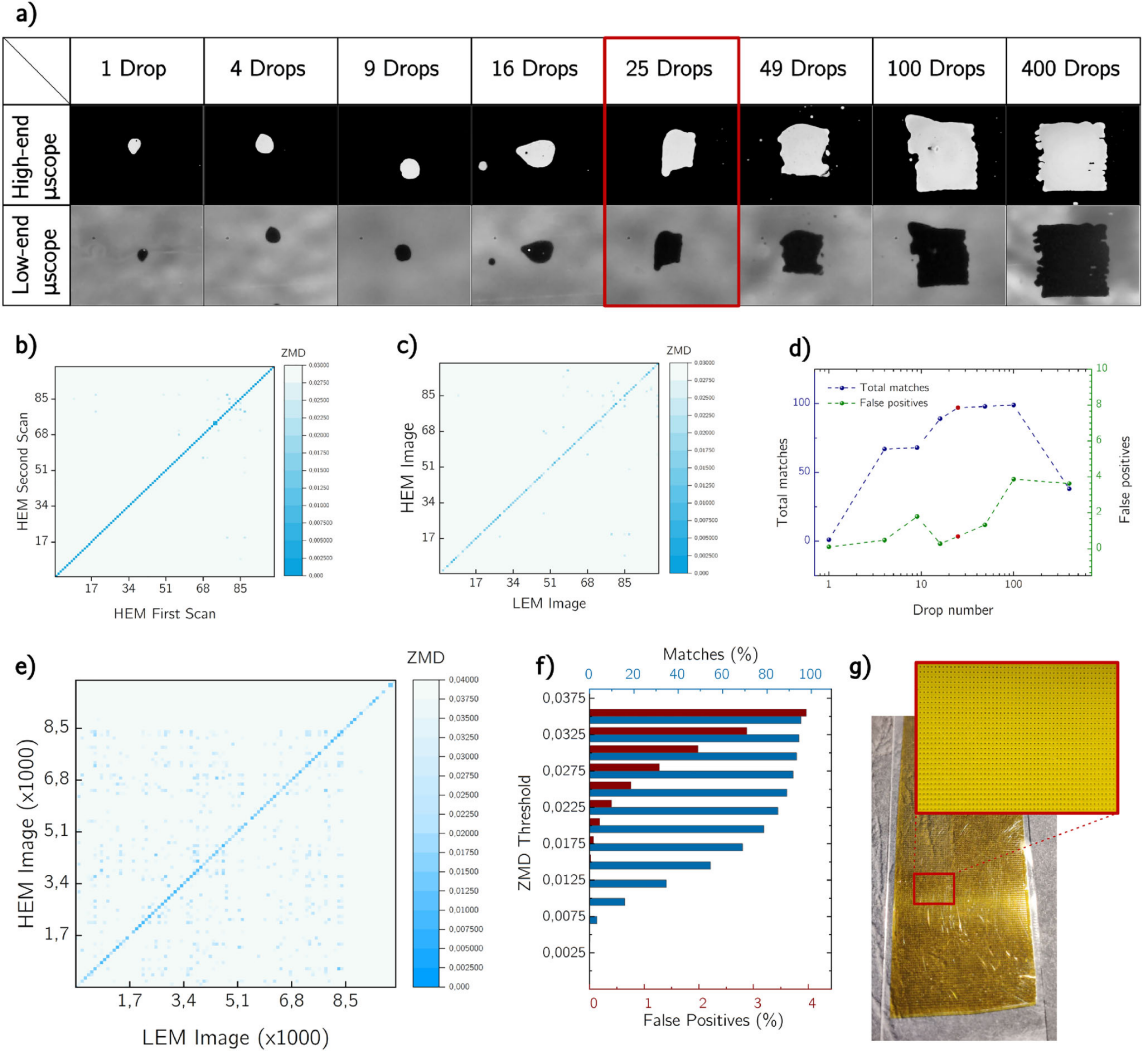
(a) Images of a series of printed PUFs with a square pattern, characterized by an increasing drop number. The acquisition has been made with two different microscopes on a set of 100 PUFs for each drop number; matching matrix for the 25 drop case, between the images taken with the HEM and itself (b) and with the HEM and the LEM (c); (d) plot reporting the variation of total matches and false positives as a function of drop number and a threshold of 0.0315 for the PUF sets in (a). (e) Matching matrix for 10,000 PUFs acquired and matched between the HEM and LEM; f) threshold analysis over the PUF images matches and false positives, with respect to the threshold; (g) overall image of the above mentioned PUF batch.

In order to achieve the maximum randomness needed for PUF uniqueness, studies on the number of drops have been carried out with the purpose to determine the optimum area of the printed PUFs, with the printing drop‐spacing always fixed at 20 μm. The experiment was carried out over a range of eight different squared PUFs with 1 to 20 drops per side, as the ones reported in Figure 2a. To investigate the possibility of PUF reading with a LEM (as in the case of using a cellphone to improve user experience), we have computed a heat‐map of the matching matrix obtained by comparing the images taken with the HEM and itself and with the LEM, with an example for the 25 drops case shown in Figure 2b,c, respectively. The color scale reported in the heat‐maps represent the ZMD, which is the geometrical distance between two moment vectors. Recognition is assumed if this distance is smaller than a predetermined threshold.

Figure 2d shows the total number of matches and the percentage of the false positive computed with respect to the different PUF areas, i.e., the number of drops. Comparative analysis among different geometries is reported in Supporting Information under Threshold Analysis, from which emerges that 25 drops geometry has a lower Equivalent Error Rate (EER) and a higher True Positive Rate (TPR) at False Acceptance Rate (FAR) of 1%. Hence, 25 drops was considered as the best choice among the others.

Moreover, from the heat map comparison of Figure 2c (25 drops case), we can see that the initial image resolution does not affect the recognition, as the total reduction in matches is about 3%, and also that using a different microscope from the user side does not cause an increase in false positives and therefore false matches. These results demonstrate that it is possible for the customer to use a different and cheaper microscope than the high‐end one used for the acquisition.

To confirm this assumption, we performed the same acquisition experiment (HEM vs LEM) for a batch of 10 000 PUFs and found that, with the same threshold, a total match rate of ≃ 95% and false positives below 2% can be achieved (see Figure 2e). Although these rates are not ideal for typical applications, it has been observed that errors in consecutive readings are uncorrelated. Therefore, applying Gaussian filters to reduce white noise in multiple LEM‐acquired images could further decrease the percentage of false positives and improve the overall match rate. An investigation of the threshold calculation is reported in Figure 2f, where the increasing trend of both matches and false positives versus the chosen threshold is clearly visible. In the Supporting Information more data about TPR and FPR for the 10 000 PUF batch are reported. In this scenario, the threshold corresponding to 1% FAR for the 25‐drop geometry is 0.0265 but has low TPR. Therefore, 0.0315 exhibits a FAR of 2.5% and an acceptable TPR that is near 95%, so that was chosen as the threshold that best strikes a balance between TPR and FAR scores. An image of the analyzed batch of 10 000 PUFs is shown in Figure 2g.

**FIGURE 3:**
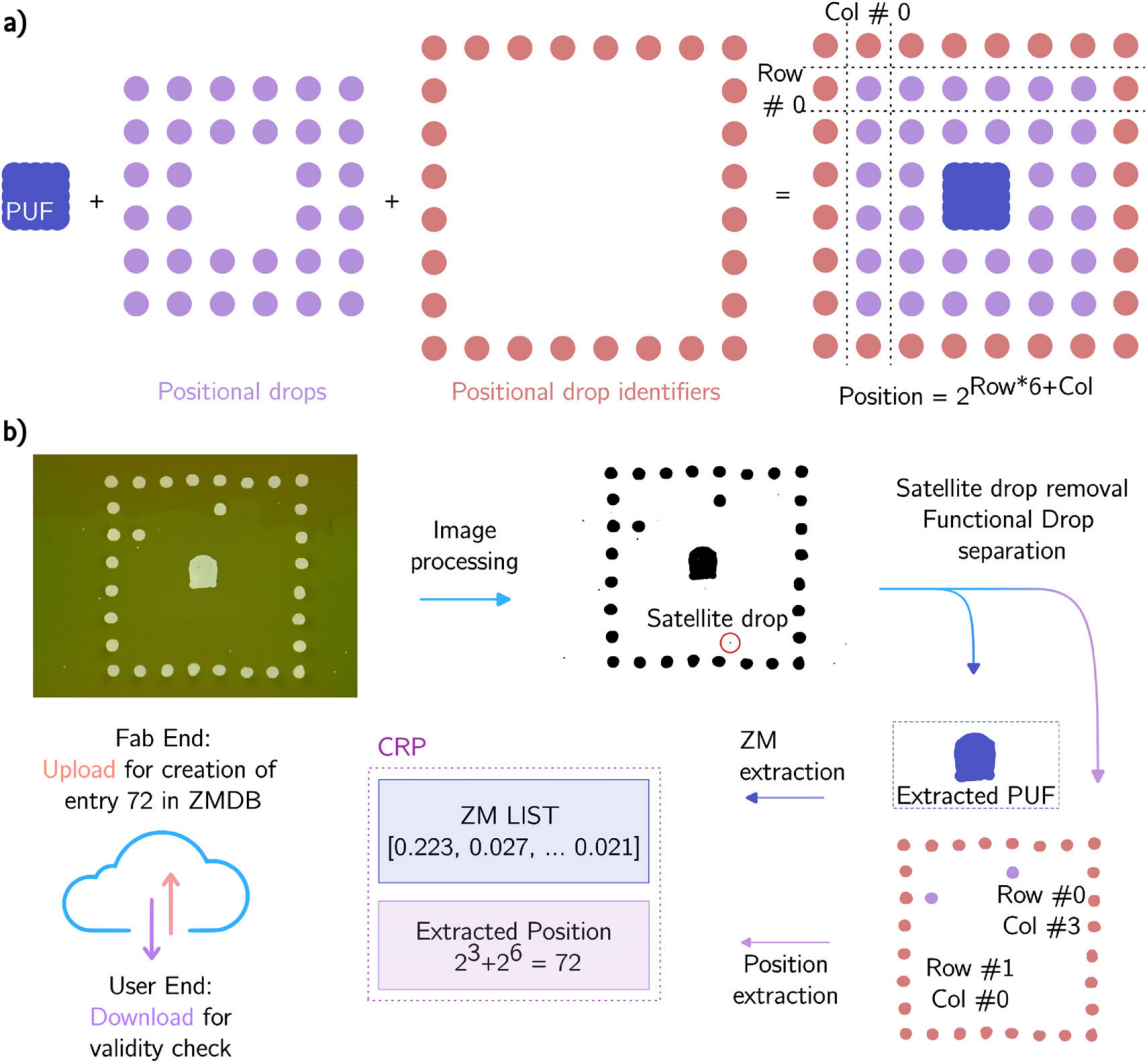
(a) Conceptual scheme of the positional recognition of a printed PUF using a surrounding positional identifier frame, which embeds the positional drops and the printed PUF; (b) procedure sequence: a single image is grayscaled and thresholded to achieve a high contrast image. Then, the script filters the satellite drops, leaving the PUF and the positional drops. The extracted ZM and the positional drops are divided and used to speed up the Challenge‐Response process.

When dealing with a large database, there may be problems with the speed of the authentication check. Our proposal to solve this problem lies in the way the ZMs are stored, i.e., the database should address the PUF in question directly when there is an incoming challenge: this allows each ZM to be associated with a PUF identifier that is easily readable by the application software. One solution could be to use a Quick Response (QR) code‐like structure, as shown in the sketch of Figure 3a, which uniquely identifies each printed PUF. In particular, the PUF (in blue) is embedded in a printed dot matrix composed of two elements: a drop position matrix (purple) and a position identification frame (pink). The pink edge drops act as position detectors for the purple internal drops, which are used to directly detect the PUF identifier, as in the following formula:

(1) $$ID_{Position}=\sum_{n}2^{6\cdot i\;+\;j}$$

where *i* is the row index and *j* the column index of the drop in purple and *n* is the number of the printed drops in the purple matrix.

The recognition process shown in Figure 3b starts with the acquisition of the original image (in color or greyscale), then it has to be filtered to create a sharp contour, which can be detected by the OpenCV *FindContours* function. At this stage, greyscale, blur, and threshold filters are applied in sequence to produce the black and white version of the image, shown in the center of Figure 3b. From this image, satellite drops are removed by area filtering, and then the internal PUF and the external code are separated by positional manipulation. The ZMs are extracted from the PUF and, in parallel, the position ID is extracted from the position matrix. For each PUF, we have a CRP, where the Challenge is the position ID and the Response is the ZM list. The CRP is now ready for storage (manufacturer end) or for direct verification against the database (user end). This identification code has proven to be highly reproducible and reliable, providing successful identification in all the cases.

### 2.3 System Performance

To underline the capabilities of our system, we have deeply characterized the PUF itself, using unicity parameters, proved robustness under physical and authentication related disturbance, discussed resistance over AI and compared the complete scalability of the system, with those in literature. Uniqueness, Reliability, Bit Aliasing and Uniformity are the most common parameters used in literature to evaluate the PUFs quality [13, 48, 49]. The Uniqueness summarizes the differences between one PUF and the others. The Bit Aliasing reports the variation over the whole PUF set of a single bit. Its optimal value is 50%, and corresponds to equal 0 and 1 probability for the value of that bit over the whole data set. Conversely, the Uniformity indicates the ratio of 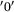 and 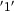 in the bit string of each specific PUF. A detailed description of how these parameters have been calculated has been reported in the Supporting Information under Section ‘Characteristic Parameters’.

We have considered three sets of PUFs with three different areas, obtained by printing 16, 25, and 49 drops per square. The parameters were then extracted from the set of images taken with both HEM and LEM. A Python script was used to extract a bit‐string from the 100 image dataset of the different PUF areas (also reported in Section PUF configuration). The bit‐string is a flattening of the part of the image obtained from the overlapping of the individual images per set: only the pixels that vary from image to image are included in the final bit‐string, excluding the background and the central part of the PUF, which is always black or white (see Section Acquisition System of the Supporting Information). The evaluated values (expressed in percentage) are reported in Table 1 and in Section Characteristic Parameters of the Supporting Information. The length of the bit‐string varies with the resolution and the area of the PUFs, as shown in the second column of Table 1. The Python script also shows that the thickness of the border decreases as the number of PUF drops increases: this means that the variability is more confined for larger PUFs, resulting in a higher number of false positives (see Figure 2). It is worth noting that for the 25 drops case, which we showed to be the optimal result in the previous section, the key metrics are uniqueness of 48.5%, bit aliasing of 41.66%, bit uniformity of 41.65%, and reliability of 99.73% demonstrating robust performance for this area as well. Moreover, extracting the bit‐string length from the images we obtain a first approximation entropy of $2^{5784}$ considering the shortest string for the chosen pattern. Our solution exhibits robustness against environmental, physical (aging, mechanical, chemical), and acquisition (resolution, rotation, translation, noise, and blur) detrimental factors. A comprehensive analysis is reported in Robustness Section in the Supporting Information. In the Supporting Information, we have added works based on inkjet printing techniques in Table S3, and a more general with other optical and solution‐processable works in Table S4. Moreover, the presented metrics, Bit Uniformity (balance of random bits within the same PUF, Intra‐HD) and Uniqueness (Inter‐HD), both exhibit values close to 50%, indicating no correlation within or between PUFs. A comparison with the results reported in literature are listed in the Section “State of the Art Comparison” of the Supporting Information. From this analysis, it emerges that our work is the only one presenting a fully scalable route from fabrication to authentication, suitable for a substantial number of production facilities. Works that report scalable fabrication techniques often face limitations due to the high cost or complexity of the associated authentication systems [15, 16, 17, 23, 25, 35, 36, 41, 43, 53, 54]. Others are constrained by fabrication methods that rely on expensive or non–industrially scalable processes [27, 32, 39, 40]. For approaches that do not fall into these categories, the main limitation arises from the storage of uniqueness features, which requires large memory databases compared to the Zernike Moments–based system proposed here [18, 19, 20, 33, 35, 38, 54, 55]. Finally, the remaining works are restricted to specific market segments and are not compatible with most luxury manufacturing processes [42].

**TABLE 1:** Key metrics of different PUFs characterized by different dimensions (16, 25, 49 drops) and acquired with two systems (HEM or LEM). The parameters, reported in percentage, are Uniqueness, the average Bit‐aliasing from each image set, the mean Reliability, and the mean bit Uniformity value extracted from the fitted distribution described in the Supporting Information, Section Bit Aliasing. The second column shows also the bit‐string length of each dataset.

	Microscope	String Length	Uniqueness [%]	Bit Aliasing [%]	Uniformity [%]	Reliability [%]
16 drops	high‐end	202955	45.8	38.88	38.88	98.81
	low‐end	5229	40.3	34.92	34.90	99.63
25 drops	high‐end	213403	48.5	41.66	41.65	96.17
	low‐end	5784	53.1	43.53	43.53	99.73
49 drops	high‐end	294035	45.8	46.55	46.55	99.16
	low‐end	7819	41.7	46.86	46.86	99.55

### 2.4 Applications

**FIGURE 4:**
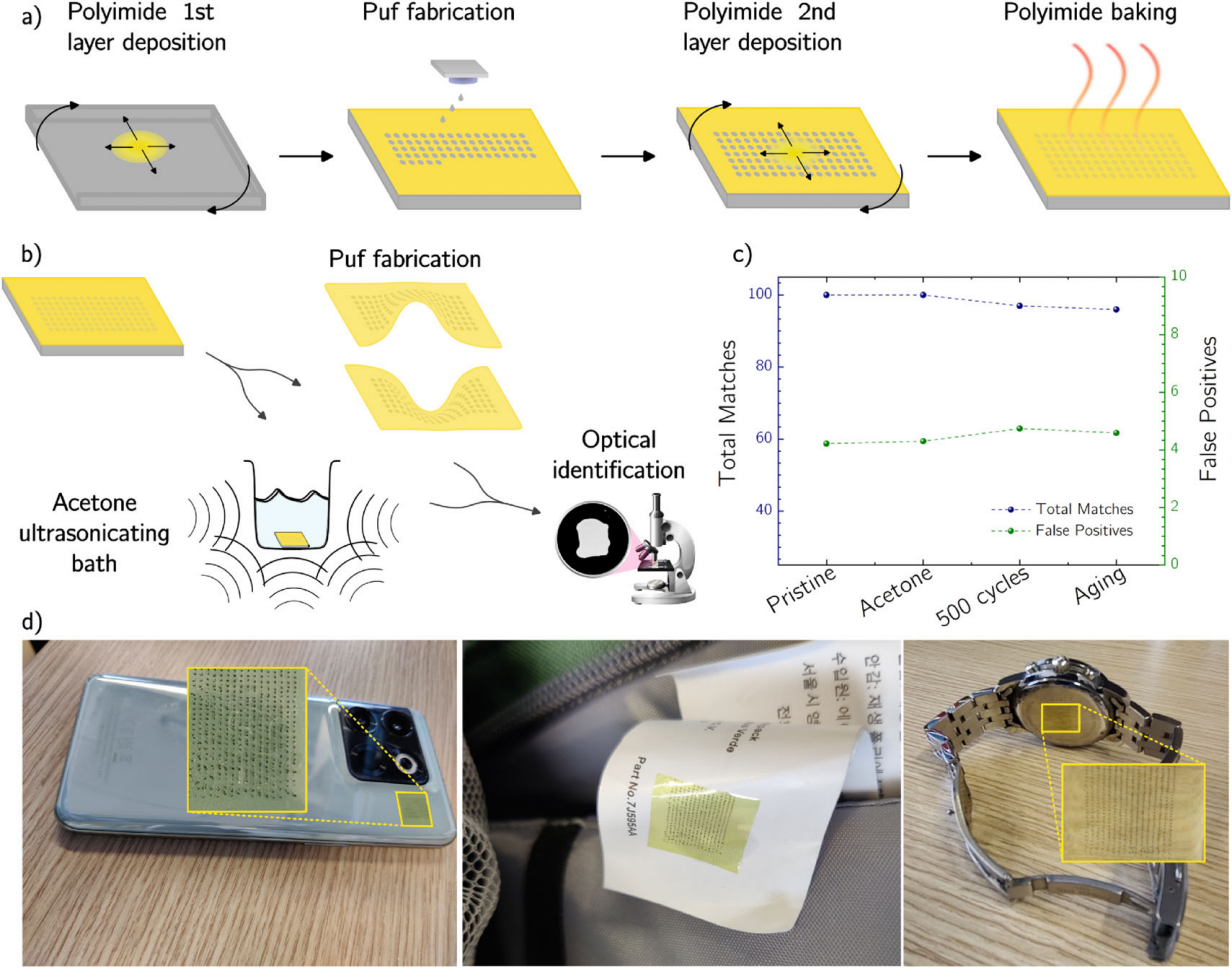
(a) Process flow for PUFs encapsulation: spin‐coating of first polyimide layer, inkjet printing of the PUF array, spin‐coating of the second layer of polyimide, baking at 300°C for 3 h; (b) chemical (sonication in acetone) and mechanical (500 bending cycles) stress tests for the PUF; (c) graph reporting total matches and false positives as a function of the consecutive stress tests; (d) possible enabled applications on luxury goods.

Luxury goods applications require high flexibility of the printed PUFs; to achieve ultra‐thin and conformable authentication tags, the printed PUFs are embedded in a double layer of spin‐coated polyimide (PI), each 3 μm thick. As shown in Figure 4a, a passivating PI layer is deposited on top of a PI substrate, where the silver PUFs are printed. Finally, a sealing bake was done at 300°C in a nitrogen atmosphere for 3 h. Mechanical properties tests have been carried out to check the bending properties and resistance to chemical agents as in Figure 4b. A comparison of the printed PUFs after 5 min sonication in an acetone bath and after 500 bending cycles shows a high chemical and mechanical resistance due to the good sealing properties of the spin‐coated PI, furthermore, experiments over aging have been carried out reporting stability after one year from the printing and encapsulation process, as reported in Figure 4c.

In Figure 4d, we show some potential applications, where a smartphone, a backpack and a watch are authenticated through ultra‐thin and conformable printed PUFs.

## 3 Conclusions

The proposed methodology establishes a comprehensive framework for the design, fabrication, and validation of inkjet‐printed physical unclonable functions PUFs. Using the inherent randomness of ink droplet deposition, this work demonstrates a scalable and cost‐effective approach to producing authentication tags that are robust to AI attacks and that can be interrogated with consumer‐grade devices. The methodology addresses key challenges, including optimizing PUF geometry for maximum uniqueness, while proposing QR‐code like mechanisms to reduce the complexity of authentication among large databases.

The fabricated PUFs exhibit robust performance, as indicated by key metrics: uniqueness of 48.5%, bit aliasing of 41.66%, bit uniformity of 41.65% and reliability of 99.73%. These metrics, derived from experimental data sets, highlight the effectiveness of the proposed geometry optimization. Furthermore, the PUFs exhibit excellent mechanical and chemical stability, retaining their structural integrity after exposure to acetone baths and repeated bending cycles. This durability makes them suitable for a variety of applications, including luxury goods authentication, supply chain security, and electronic device tagging.

The integration of a low‐cost imaging system, such as a smartphone with a zoom lens, further enhances the accessibility and practicality of the proposed solution, while ensuring accurate and reliable identification.

## 4 Experimental Section

### 4.1 Fabrication of the Inkjet Printed PUFs

A polyimide layer(PI2611, purchased from HD Microsystems) was spun on a silicon wafer. On top of it, the PUF squared patterns were printed with the commercial printer Fujifilm Dimatix DMP2850 with a silver ink (Sigma Aldrich, art. num. 736465). For those applications with biocompatibility constraints, the silver ink can be substituted with biocompatible material‐based inks characterized by a sufficient contrast with the substrate in use. As example, PUFs made with PEDOT:PSS are reported in the Supporting Information. A soft drying step (50°C for 5 min) was applied before the spinning of the second polyimide layer. Finally, the stack was baked at 300°C in nitrogen atmosphere for 3h.

### 4.2 Data Acquisition

The stitching of the printed PUF matrix was taken with two different cameras, the high‐end optics of the microscope LEICA DVM6a and a low‐end optical microscope (Juision 40x‐1000x). The first had an embedded stitching software to acquire images on the whole array, the second, instead was mounted on a custom prototype, and a Python script running on the controller of the prototype allowed to perform the stitching of the images. In both cases, the images were preferably taken in greyscale format, otherwise, if not possible (the Juision has only color format) the images were converted to greyscale.

## Conflicts of Interest

Some authors are affiliated also with Quantavis s.r.l., a spinoff company of the University of Pisa in the business of developing advanced anticounterfeiting solutions based on semiconductor technology and on advanced materials.

## Supporting information


**Supporting File**: smll73467‐sup‐0001‐SuppMat.pdf.

## Data Availability

The data that support the findings of this study are available from the corresponding author upon reasonable request.
