# Supplementary material for: Inkjet‐Printed Physical Unclonable Functions For Secure Authentication
Source: Small. 2026 Apr 30;22(33):e14908. doi: 10.1002/smll.202514908 (PMC13262241; doi:10.1002/smll.202514908)
Supplement: Supplementary file 1 — Supporting File: smll73467‐sup‐0001‐SuppMat.pdf. [file SMLL-22-e14908-s001.pdf]

Inkjet-Printed Physical Unclonable Functions for Secure Authentication - Supporting Information

Riccardo Sargeni,[1,2] Elisabetta Dimaggio,[1] Francesco Pieri,[1]
Stefano Di Pascoli,[1] Giuseppe Iannaccone,[1,2] and Gianluca Fiori[1,*]

[1] *Dipartimento di Ingegneria dell' Informazione, Università di Pisa, Pisa, Italy*
[2] *Quantavis s.r.l., Largo Padre Renzo Spadoni, Pisa, Italy*

(Dated: March 9, 2026)

CONTENTS

Acquisition Systems 2

Threshold Analysis 3

Image scaling factor 7

Characteristic parameters 8
- Uniqueness 10
- Reliability 10
- Bit Uniformity 10
- Bit Aliasing 12

Robustness 12

Adaptability on other substrates 16

Biocompatible ink analysis 16

Random geometry study 17

State of the Art Comparison 18

References 21

ACQUISITION SYSTEMS

The acquisition systems considered for the PUF images are a high-resolution one and a low-resolution one. The high resolution system is based on a high-end optics mounted on a commercial microscope, the LEICA DVM6A. The LEICA software provides a stitching mode, which allows to have a fast acquisition of a large number of PUFs, as the 10000 reported in the **PUF configuration** section of the main manuscript. At this stage, each image is acquired with a resolution of 1600×1200 pixel, with a Field Of View (FOW) that varies according to the PUF size: 400×300 μm^2 for dimension up to 49 drops per area, $600 \times 450 \mu m^2$ for 100 and 400 drop count and 960×720 μm^2 for the acquisition over the positional matrix. To save memory and skip a grayscale conversion, required for image processing, images are also captured directly in grayscale format. The low-resolution system, instead, relies on a low-end optics (Juision 40x-1000x) mounted on a high-precision manufacturing prototype [1], which allows to acquire multiple images thanks to a high-resolution stepper motor. A custom python script, running on the controller of the prototype, allows to have a stitching of the whole matrix, taking into account also the sample alignment. Here, the resolution is fixed at 640×480 pixels, and the focus can be turned ON and OFF, achieving a maximum pixel size of $\simeq 1.5\mu m$/pixel when the zoom is ON. The images acquired with this system have to be converted in grayscale and, when needed by the array pitch, cut to include only a single PUF per image. Figure S1a-b show two pictures of both the high and low resolution acquisition systems, respectively.

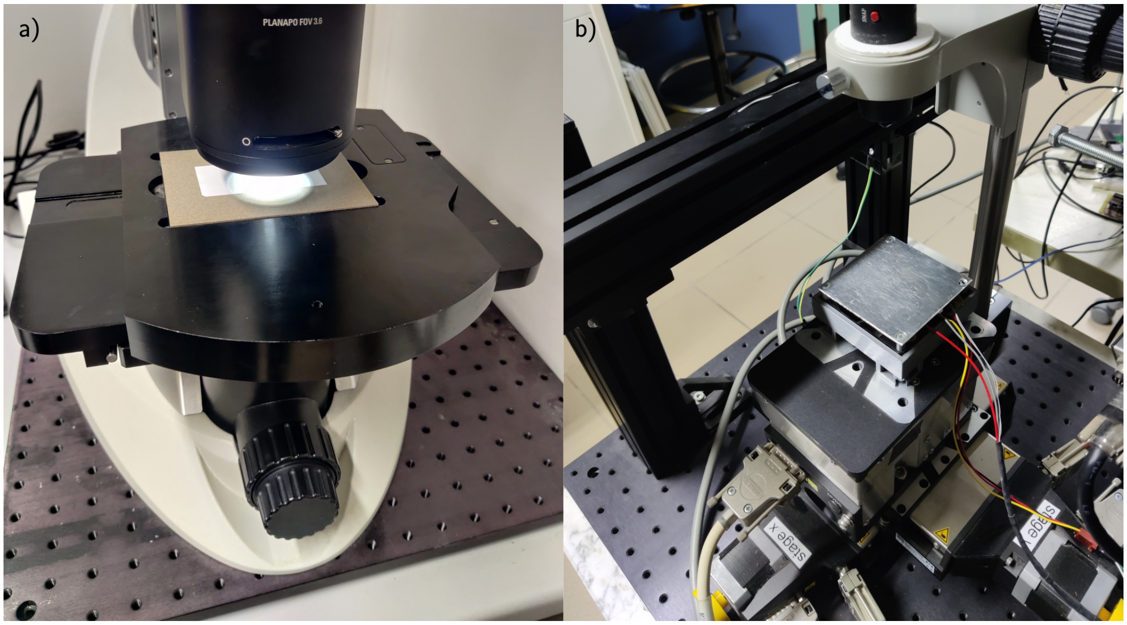

Figure S1. Pictures of a) the high resolution acquisition system based on the LEICA microscope optics; b) the in-house high-precision and low-resolution acquisition system.

THRESHOLD ANALYSIS

In this section, we report the analysis performed to find the impact of the authenticity threshold value on the number of matches and of the false positives. In particular, for each drop count, the images acquired with the high-end and low-end optics have been processed founding an increasing trend with the number of drops, hence of the PUF area, in both matches and false-positives. It appears from the Graphs in Figure S2a and b that the matching curves have a sigmoidal trend (see Equation S1), while False Positive curves are exponential. The fitting parameters, referred to the Equations S1 and S2, are reported in table S1.

$$\sigma(x) = \frac{B}{1 + e^{-K\ (x-C)}} \tag{S1}$$

$$exp(x) = y_0 + A \cdot e^{Rx} \tag{S2}$$

As remarked in the main text, from this analysis it emerges that to minimize the number of false positives maintaining a higher number of matches, the optimal choice is a 25-drop PUF with a threshold between 0.03 an 0.0325, which has been set at 0.0315. In Figure S3 and S4, the fitted curves for 1 a), 4 b), 9 c), 16 d), 25 e), 49 f) 100 g) and 400 h) drops and

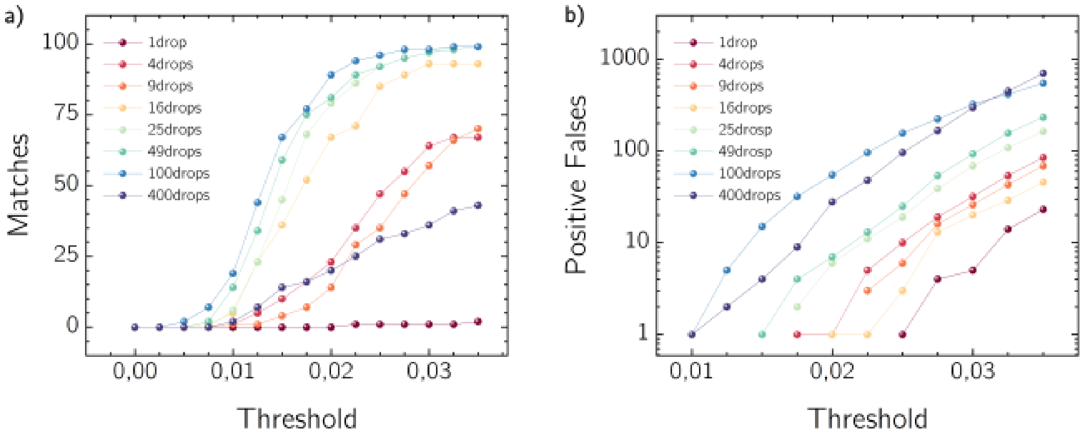

Figure S2. Matches (a) and false positives (b) versus the authenticity threshold for an increasing number of drops, hence of PUF area.

	y0	A	R	B	C	K
1 drop	–	–	–	2	–	0
4 drops	-1,0832	0,08542	197,95393	70,90547	0,02251	264,87352
9 drops	–	–	–	74,99024	0,02529	262,90396
16 drops	-0,91275	0,06472	188,63261	92,89795	0,01695	295,8922
25 drops	-2,54318	0,25736	185,48009	96,27555	0,01556	392,44129
49 drops	-3,34401	0,30445	190,79259	95,44428	0,01417	391,20801
100 drops	-24,59504	8,79418	120,00568	97,56959	0,01333	388,23953
400 drops	-9,2315	1,13431	184,20791	43,88517	0,02084	200,57607

Table S1. Fitting parameters for the curve extracted from matches and false positives varying the authenticity threshold. Each image set, taken with respect to the PUF dimension, has been fitted with the equation S1-S2 .

the Receiver Operating Characteristics (ROC) curve are respectively reported.

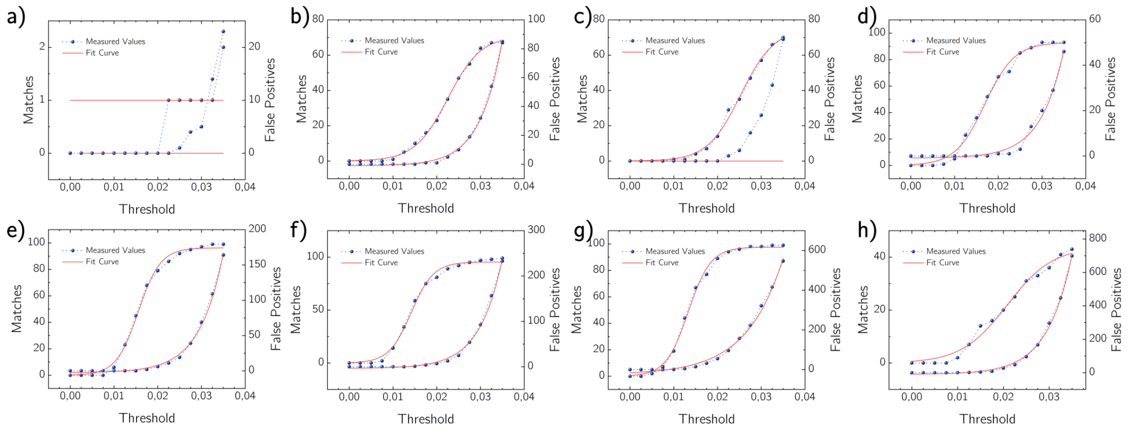

Figure S3. Images of the trend for matches and false positives with respect to the threshold for 1 a), 4 b), 9 c), 16 d), 25 e), 49 f) 100 g) and 400 h) drops.

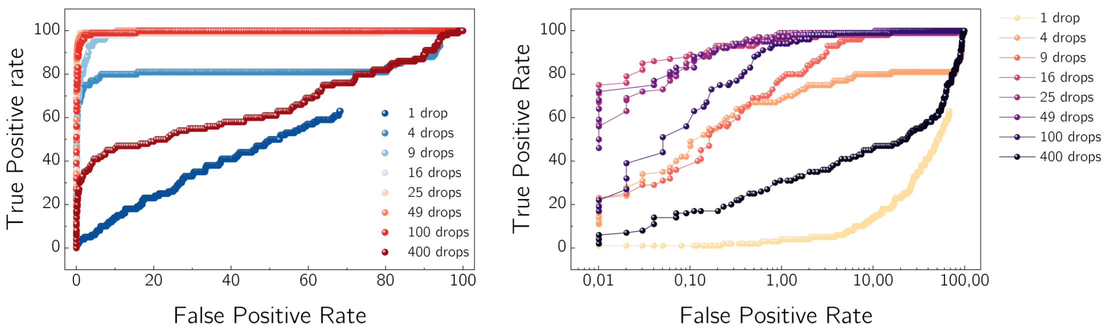

Figure S4. (a) linear scale and (b) log scale of ROC curves for matches and false positives varying with the chosen threshold and the PUF dimension. As visible in the log scale graph the 25 drops PUF reaches an higher value of matches for less positive falses.

FPR (%)	TPR (%)	Threshold	Note
1.064543	91.48	0.0265	TPR @ FAR 1% = 91.5%
1.297530	92.46	0.0275	
1.561024	93.23	0.0285	
1.855364	93.84	0.0295	
2.181182	94.26	0.0305	
2.536375	94.67	0.0315	TPR @ FAR 2.5% = 94.5%
2.925356	95.14	0.0325	
3.344003	95.58	0.0335	
3.794304	95.95	0.0345	EER < 4%

Table S2. ROC operating points at different decision thresholds. Rows with note indicate relevant security metrics (TPR at fixed FAR and EER region).

IMAGE SCALING FACTOR

To have robustness over differently scaled images, image resizing must be performed before the extraction of the Zernike moments. As reported from Chong et al.[2], scale transformation affects the Zernike moments by a scaling factor β that can be derived from the following formula, where m'_{pq} is the generic moment of order p and degree q:

$$m'_{pq} = \iint_{\mathcal{P}} x^p y^q f\left(\frac{x}{a}, \frac{y}{a}\right) dx\ dy = \iint_{\mathcal{P}} a^{p+q+2} \xi^p \eta^q f(\xi, \eta)\, d\xi\ d\eta = a^{p+q+2} \cdot m_{pq} \tag{S3}$$

We compute m_{00} and then choose a such that m'_{00} is a preassigned value β, independent of the resolution of the original image. It is easy to show that $a = \sqrt{\beta / m_{00}}$ must hold. Smaller values of β result in a smaller computational burden, but a larger information loss. A graph reporting the trend of matches and false positive as a function of the chosen β (PUF area in x-axis) is reported in Figure S5.

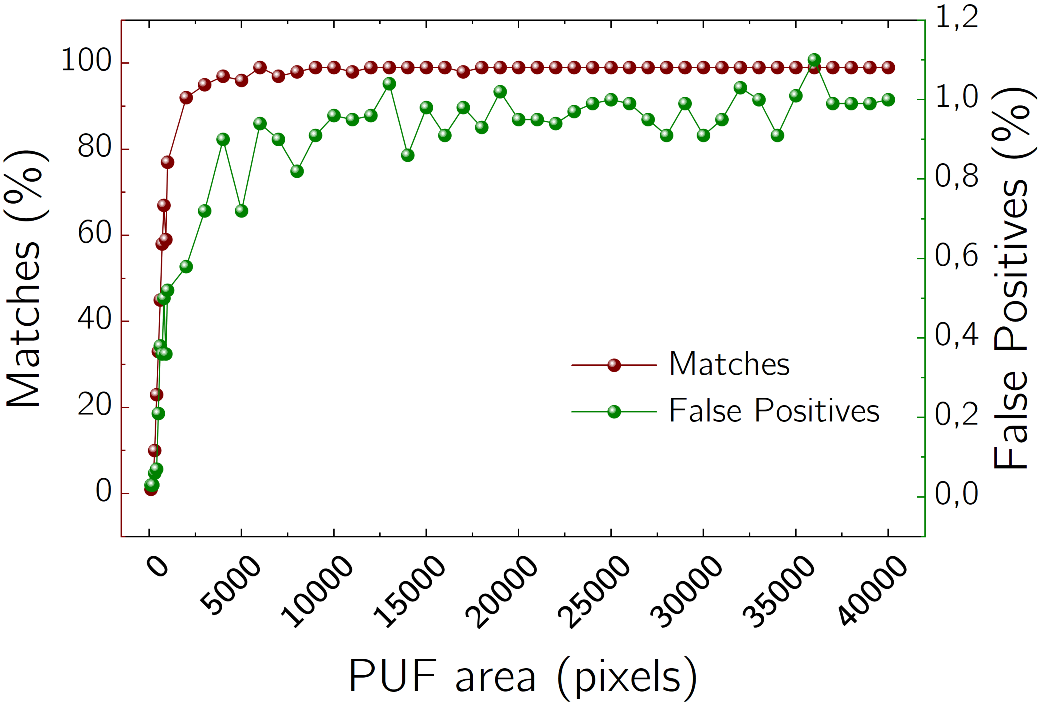

Figure S5. graph reporting the trend of matches and false positives as a function of the PUF area used to image rescaling.

CHARACTERISTIC PARAMETERS

From an analytical point of view, a PUF can be characterized by several parameters that explain how its use can be robust and reliable in a real scenario. In this paper, we have focused on the parameters that are mainly considered in the literature in order to have a fair comparison: uniqueness, bit uniformity and bit aliasing [3–5]. The evaluation of these parameters requires the assignment of a unique bit string to each PUF.

Our procedure to extract a unique bit string for each PUF is shown in Figure S6, and it is divided in three main steps:

- Image Processing;
- Logical Operations;
- Bit string extraction.

The procedure was applied to all the image datasets collected, one for each PUF matrix (1 to 400 drops). Fig. S6a shows the image processing step in detail: starting from the acquired dataset, the images are processed using the Python module OpenCV, which applies a first grayscale and blur transformation, followed by an automatic black and white thresholding (Otsu method [6]). Then the images are overlapped to extract a *Zone Mask*, which is characteristic of each dataset and that is used in the Logical Operation step.

Fig. S6b describes the logical operations applied on the processed dataset. At first, the centroid of each image and of the *Zone Mask* are extracted. The image centroids are needed to align the images to the coordinates of the *Zone Mask* centroid, which is the starting point for a logical AND and OR; so, each image is spatially translated to place its centroid in the same position as the *Zone Mask* centroid; in this way, the logical AND/OR operations result in the minimum contour obtainable with the overlap. The AND and OR operations are applied among all the centered images of the dataset, and followed by a logic XOR between the two resulting images. The final image shows a portion of area containing the information of all the contours of the dataset.

Finally, the bit string is extracted as shown in Fig. S6c :

- the indexes of the white pixels ('1') of the *Contour Mask* are extracted;

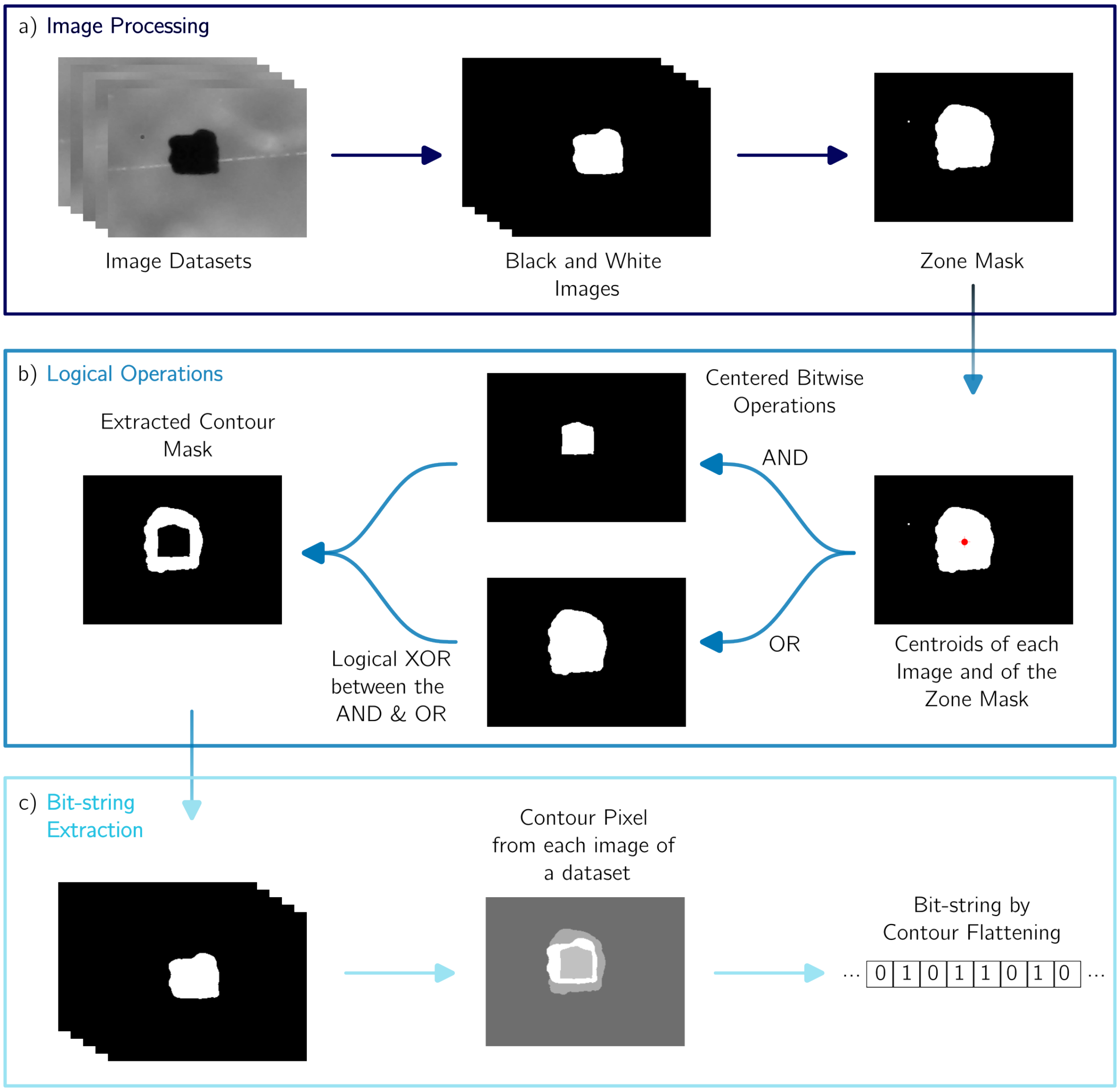

Figure S6. Image processing to extract a bit-string from each PUF contour. a)Image processing for the Zone mask extraction; b) Logical operations for the contour mask extraction; c) bit string extraction.

- these indexes are used to extract the corresponding pixel values('0' if black or '1' if white) from each centered image of the dataset and to fill in an array;

- the array is vectorized using the numpy *flatten* attribute, considering the same bit string length for each image. The bit string is also positionally associated with respect to the centroid calibration.

Uniqueness

Uniqueness is the parameter that indicates the degree to which the responses generated by a PUF are unique across all the different PUFs. It is calculated with equation S4:

$$\text{Uniqueness} = \frac{2}{K(K-1)} \cdot \sum_{i=1}^{K-1} \sum_{j=i+1}^{K} \frac{HD\ (\ R_i(n)\ ,\ R_j(n)\ )}{n} \cdot\ 100\ \ [\%] \tag{S4}$$

where K is the total number of PUFs, i, j are the PUF indexes extracted from the dataset, and n is the length of the bit string. The optimal value for this parameter is around 50%, corresponding to a perfect randomness in the pixel value between the bit strings.

Reliability

Reliabiltiy refers to the robustness in the recognition mechanism.

$$Reliability = 100\ -\ \frac{1}{K} \cdot \sum_{i=0}^{K} \frac{1}{T} \sum_{l=0}^{T} \frac{HD\ (\ R_i^0(n)\ ,\ R_i^l(n)\ )}{n} \cdot\ 100\ \ [\%] \tag{S5}$$

In **Equation S5**, K is the total PUF number, T is the total number of trial, i and l are respectively, the index of a generic PUF and the different trials for the same PUF, n is the length of the PUF string. In our case the total trial number is 5 and the total PUF number considered is 100. Different trials are made in sequence for each microscope, to consider the robustness of our recognition method to white noise deriving from different acquisitions.

Bit Uniformity

Bit Uniformity is the proportion between the number of '0' and '1' in the same PUF bit-string. It is calculated with the equation S6:

$$\text{Uniformity} = \frac{1}{N} \cdot \sum_{l=1}^{N} R_{i,l}\ \cdot 100\ \ [\%] \tag{S6}$$

where N is the length of the bit string, and l is the index referring to the single bit of the i-th PUF. As for the uniqueness, the bit uniformity optimal value is 50% , which means having a homogeneity between *'0'* and *'1'* in the bit-string. Figure S7 shows the distribution of

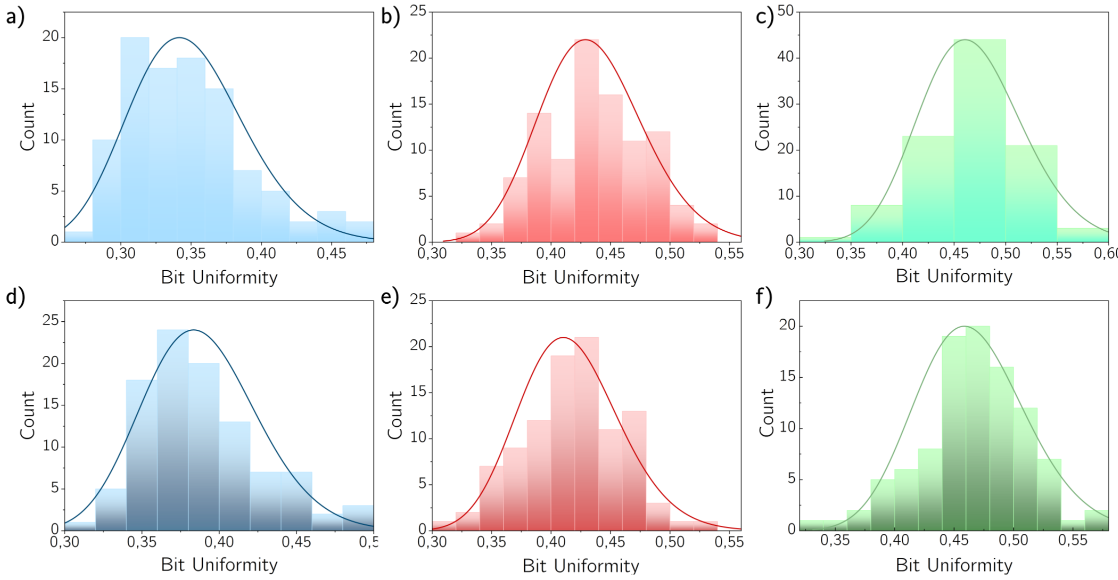

Figure S7. Hystograms of printed PUFs over 100 image datasets, taken on 16 (blue), 25 (red) and 49 (green) drop area PUF with two different microscopes: a, b, c high-end, d, e, f low-end.

	Microscope	Mean	Std dev	L 95% CI	U 95% CI	Min	Med	Max
16 drops	high-end	0,38882	0,0382	0,38124	0,3964	0,31621	0,38318	0,49238
	low-end	0,349	0,04265	0,34054	0,35747	0,26563	0,34242	0,46261
25 drops	high-end	0,41665	0,04035	0,30838	0,42491	0,31573	0,38607	0,5239
	low-end	0,43536	0,04116	0,42679	0,44394	0,33904	0,43646	0,53475
49 drops	high-end	0,46551	0,04511	0,45656	0,47446	0,32491	0,46833	0,57218
	low-end	0,46861	0,04912	0,45886	0,47835	0,31296	0,46937	0,58959

Table S3. Descriptive statistics for the bit uniformity on two times acquired images sets of 100 PUFs with 16, 25 and 49 drop per area. Mean, standard deviation, upper and lower 95% confidence intervals, minimum, maximum and median values are reported for the extracted bit uniformity for each image set. Entries labeled as "high-end" and "low-end" refer to the images acquired with LEICA microscope and the low-end microscope.

bit uniformity for the images of each analyzed dataset. All the fitting parameters for that distribution are resumed in Table S3.

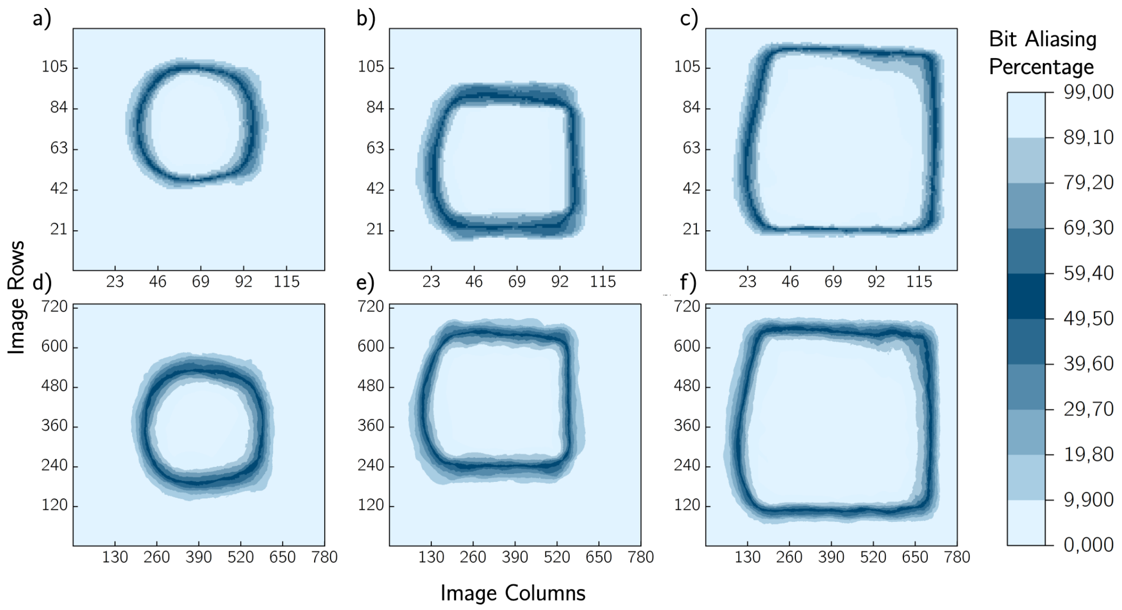

Figure S8. Images reporting the border of the analyzed PUFs with different size, 16 (a-d), 25 (b-e) and 49 (c-f) drop area count) and microscope, low-end a-c) and high-end d-f)

Bit Aliasing

Bit Aliasing statistics reports the variation of a single bit over the entire PUF dataset bit-strings, so a bit aliasing of 50% is the optimal value, which is a index of chaotic variation for the same bit among the whole data set. It is calculated with the equation S7:

$$Bit\ Aliasing = \frac{1}{K} \cdot \sum_{i=1}^{K} R_i(j) \ \cdot \ 100 \ \ [\%] \tag{S7}$$

In **Equation** S7, K is the total amount of PUF, i is the PUF index over the series, j is fixed and refers to the j-th bit. Figure S8 reports a heatmap for the 16, 25, 49 drops datasets of the pixel distribution in the Contour Mask region.

ROBUSTNESS

To evaluate the robustness of the proposed system, we analyzed the True Positive (match) and False Positive scores under several conditions. Since degradation can occur due to both environmental and acquisition-related factors, these scores were extracted and compared across all tested conditions.

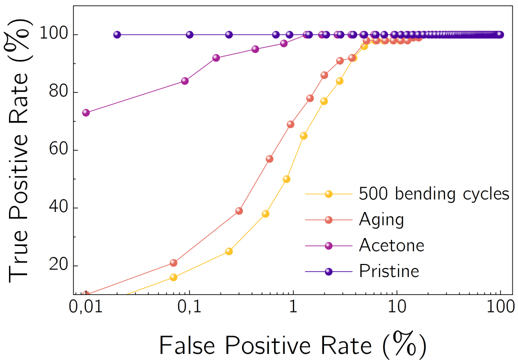

Figure S9. comprehensive ROC curves for passivation analysis of Figure 4c, as function of threshold and for different stress conditions.

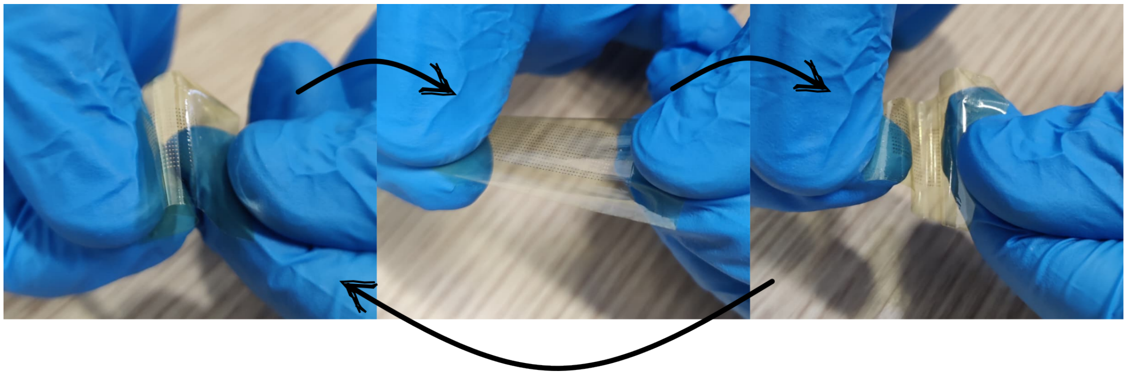

Figure S10. Representation of how a bending cycle was performed.

For each condition, the analysis was performed by recording the number of matches and false positives in both unperturbed and perturbed scenarios, as a function of the decision threshold. In the case of perturbations related to the acquisition process (software-level perturbations), the unperturbed HEM-acquired images were compared with the perturbed LEM-acquired images, and the resulting scores correspond to the performance of this identification test.

Environmental Perturbation: In this case, analyses of chemical, mechanical, and stability robustness were performed. The polyimide (PI)-encapsulated set of PUFs was photographed immediately after fabrication, which included a baking step at 300 °C for 2 hours, and this condition is reported as Pristine in Figure S9. Subsequently, the same samples were subjected to a 5-minute ultrasonic bath in acetone to assess chemical resistance, followed by 500 bending cycles to evaluate mechanical durability, as shown in Figure S10

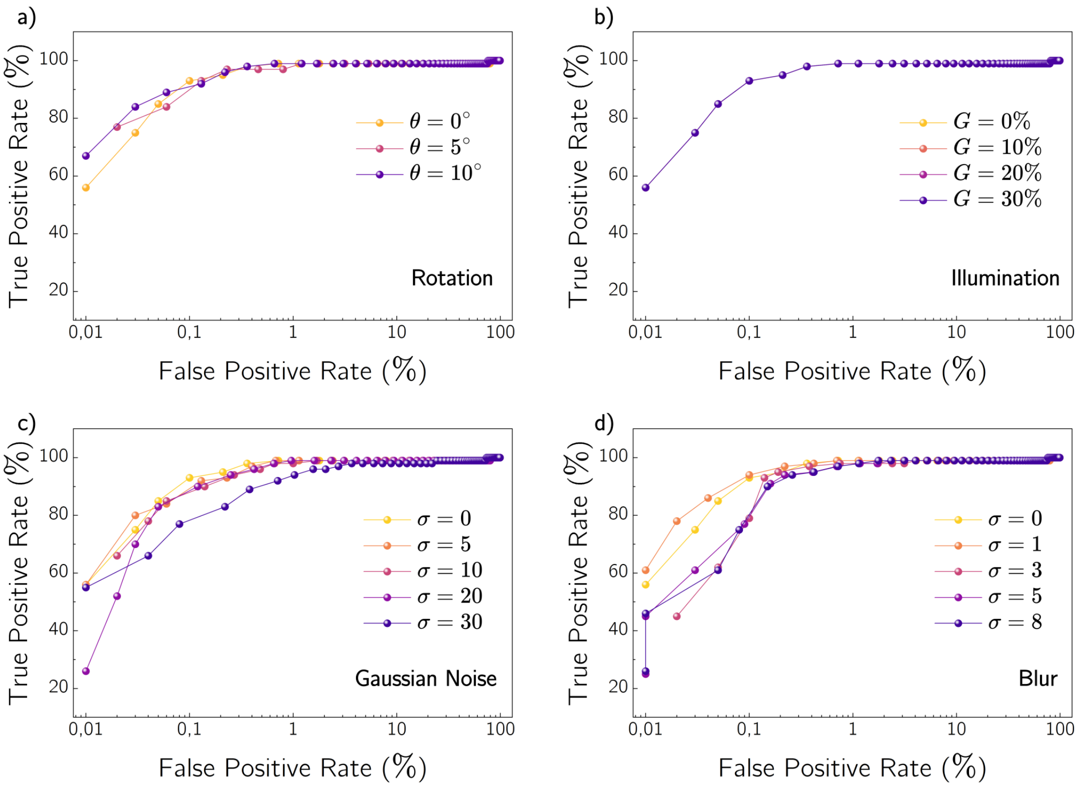

Figure S11. ROC curves analysis for different image perturbations intensity. a) reports robustness for three different angles rotation (0°,5°,10°); b) reports ROC curves for 4 illumination Gain (0%, 10%, 20%, 30%); c) reports an added Gaussian Noise for 5 different values of standard deviation (0, 5, 10, 20, 30); d) reports ROC curves for blur perturbation of 5 different sigma values intensity (0, 1, 3, 5, 8), with a given 5x5 blurring matrix.

After 11 months the same PUF sets has been re-photographed to assess the long-term stability. Results are all reported in Figure S9.

Image Perturbation: Variability in the acquired images was analyzed through software-based modifications. Rotation invariance is a well-known property of Zernike Moments (ZMs) when the phase component is discarded; however, Figure S11a provides further confirmation of this invariance for rotation angles of 5° and 10°. Gaussian noise was added using the normal function of the NumPy Python module:

noise = rng.normal(mean, sigma, size=img.shape).astype(np.float32)

where sigma is the standard deviation representing the perturbation level, as shown in

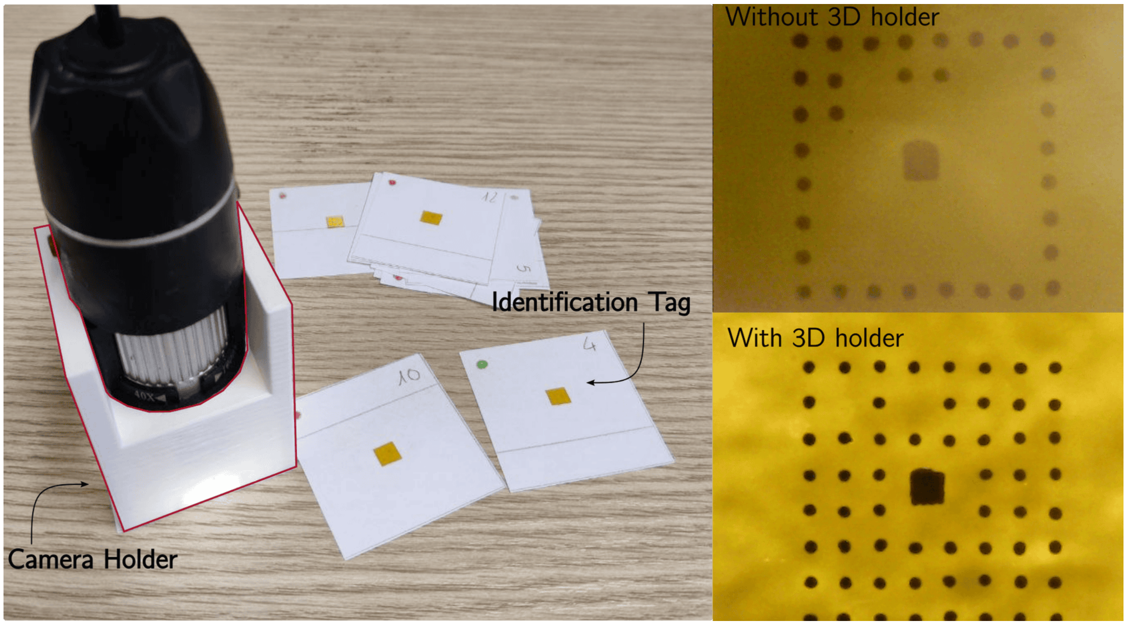

Figure S12. developed demonstrator with the added 3D printed holder, which gives constant illumination and a fixed focus value for the final authentication.

Figure S11c. Similarly, blurring perturbation was applied using the GaussianBlur function from the OpenCV module, configured as:

$$\text{blurred} = \text{cv2.GaussianBlur}(\text{gray}, (5, 5), \text{sigma})$$

where sigma is the perturbation parameter and (5, 5) defines the convolution kernel size. A particular consideration must be given to illumination. The gain value (G) reported corresponds to the percentage used to enhance the pixel intensity of individual cells and does not significantly degrade PUF performance. It is already known that a certain level of illumination is necessary to achieve sufficient contrast between the PUF and the background, especially when using Otsu's thresholding. A 3D-printed holder has been developed (see Figure S12) to ensure uniform lighting and maintain a fixed focal distance between the camera and the tag, thereby improving acquisition consistency during authentication.

ADAPTABILITY ON OTHER SUBSTRATES

Experiments on additional substrates are reported in Figure S13, where a comparison is made among glass, paper, and plastic substrates. All samples were fabricated in batches of 100 PUFs with 25 drops area. The results indicate that glass and untreated paper are more promising than treated paper and Nitto tape (plastic film) in terms of maximum Area Under Curve (AUC).

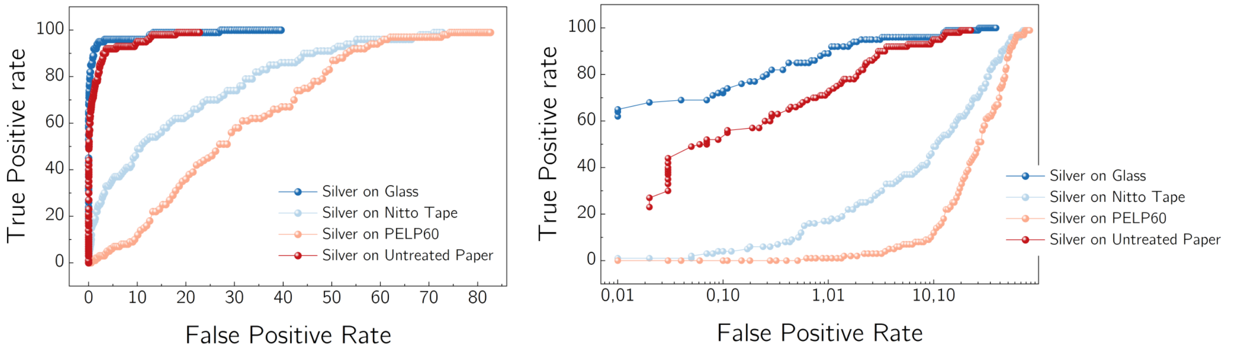

Figure S13. ROC curve in linear and log scale of PUFs fabricated on Glass, Nitto Tape, PELP60 (special paper) and untreated paper.

BIOCOMPATIBLE INK ANALYSIS

PEDOT:PSS has been exploited as a different ink squared geometry PUFs on polyimide substrate. We have performed a test on a batch of 100 PUFs acquired in successive steps with the Leica DVM6 microscope, as reported in Acquisition System section. Results are summarized in the ROC curves reported in Figure S14 , together with the scan stitch of the samples.

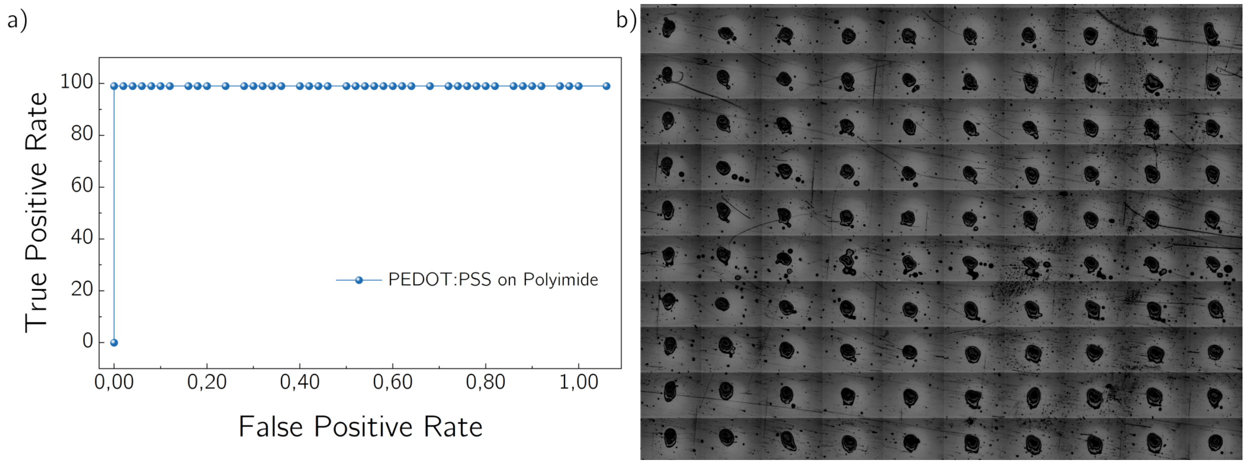

Figure S14. a) reports the ROC curves for the PEDOT:PSS printed PUF on polyimide substrate; b) reports the image stitch of the 100 PUFs set acquired in this experiment.

RANDOM GEOMETRY STUDY

To assess the applicability of our recognition mechanism with different printed geometries, we have printed a random pattern on PI film, made with silver ink arranged in a matrix of 7x6 pixel, filled with 25 drops randomly positioned. Results are reported in Figure S15, which shows a photo of the printed matrix, the linear and log scale of the ROC curves, and the trend of matches and false positives *vs* threshold: the TPR @ 1% of FAR is equal to 96% and we have a Equivalent Error Rate (EER) = 3% for th_{EER} =0.061

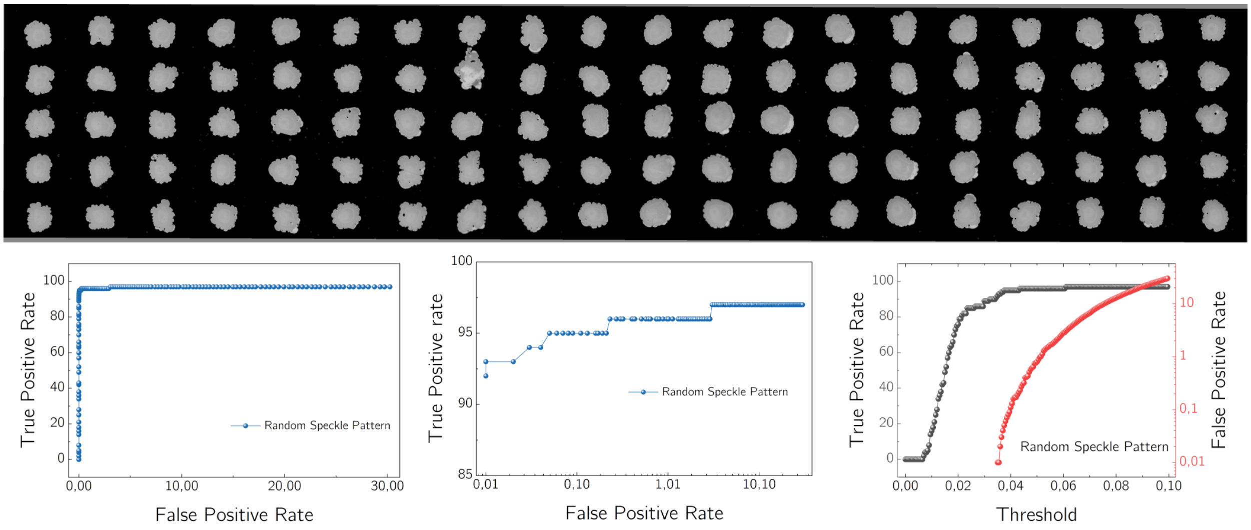

Figure S15. Figures reports the overall image of the random speckle pattern, and the results obtained for the True Positive Rate and False Positive Rate reported in the ROC curves (linear and log scale) and as a function of the threshold.

STATE OF THE ART COMPARISON

In this section, comparison tables between our work and other similar works referring to optical and/or inkjet printed PUFs are reported.

Ref.	Fabrication Tech	Dimension	Unique Feature	Acquisition Facility	Feature extraction	Validation	Robustness	Missing Analysis/robustness lack
Our Work	Inkjet Printing	<100×100 μm^2	Contour of printed feature	Low cost camera (<50$)	ZM transform	Threshold between ZM distance	Chemical, mechanical, long-term stability, resolution, rotation, translation, noise, blur	Light
[7]	Inket printing	Undeclared but likely >300×1000 μm^2	Optical variability of printed inks	High resolution microscope	Bit-string generated for dowscaled image	Bit-string comparison	-	Downscaling, rotation, alignement, light, noise, long-term stability, chemical, mechanical, etc etc.
[8]	Reverse-offset printing + inkjet printing	300×400 mm^2	Conduction probability of nanowire networks	Electrical measurement	String of 25 bit ('1' shorted, '0' open)	Bit-string comparison	-	No robustness analysis is reported
[9]	Inkjet printing	>1 cm^2	Reflectance spectra of printed features	High cost microscope	Reflectance spectrum of the acquired image	Reflectance spectrum comparison (colour validation), AI validation	Water robustness (after treatment), mechanical robustness	Long-term stability, resolution, chemical stability, noise, rotation traslation
[10]	Inkjet-printing	Undeclared	Self-assembly of colloidal photonic crystals	High cost fluorescence microscopy	Reflective pattern + fluorescence pattern	Ai validation based on spectral analysis	-	Lacking robustness study
[11]	Spin-coating + inkjet printing	>1 cm^2	Fluorescence QD's patterns	High cost fluorescence microscopes	Fluorescence spectrum	AI-based fluorescent pattern check	-	Lacking robustness study
[12]	Inkjet printing	>1 cm^2	Color pattern	Smartphone	Optical Image	Watermark extraction from optical image	Colour distortion, rotation, traslation, edible	Downscaling, long-term stability, light, chemical, mechanical, noise.

Table S4.

Reference	Uniqueness	Reliability	Bit Unif.	Bit Al.	Encoding Cap	Fabrication	Acquisition	Authentication
This Work	48.5	99.73	41.65	41.66	$\geq 2^{2659}$	Inkjet Printing, low-cost, flex	Optical Acquisition	Contour Check, reproducible with costly machinery
[13]	-	-	-	-	-	Metallic ink Dropcasting, low cost	Light Diffraction	Pattern matching
[7]	-	-	-	-	-	Inkjet Printing, low-cost	Image Acquisition	Contour check, reproducible with costly machinery
[14]	-	-	-	-	-	Lithography, high-cost	Image acquisition	AI assisted check, reproducible with costly machinery
[15]	-	-	0.49	-	-	Screen Printing, low-cost,flex	Image Acquisition	Image Comparison
[16]	-	-	-	-	-	Inkjet Printing, low-cost, flex	Optical Acquisition	Color comparison
[17]	45.93	99.15	-	35.89	2^{45}	Third Part Structures, flex	Optical, Spectroscopy, Electrical	Three key check
[8]	up to 50	-	-	-	-	Reverse Offset, Inkjet, medium-cost, flex	Electrical	Short-Open Circuit
[18]	-	-	-	-	-	Printing, low-cost, flex	Electrical	Resistance Value
[9]	-	-	-	-	$\simeq 2^{2659}$	Inkjet Printing, low-cost	Optical Acquisition	AI assisted check
[19]	-	-	-	-	-	Electrochemical Exfoliation, Dropcasting, low-cost, flex	Image Acquisition	Contour Check
[20]	0.454	0.805	0.481	-	$\simeq 2^{288}$	Spin Coating, Laser Printing, medium-cost, flex	Optical, Electrical	AI assisted check
[21]			0.4997		2^{10000}	Spin Coating, low-cost, flex	Optical	ROI laser scattering
[22]	48.1	97.67	48.4			Spin Coating, low-cost, flex	Electrical	Memristive value, resistant to machine-learning attacks
[23]	49.8	93	-	49.2	2^{217895}	Nanoprinting, high-cost	Photoluminescence	AI assisted check, resistant to nanolithography and nanomoulding
[24]	50.06	91.27	50.02		-	Stirring, Baking, low-cost, flex	Optical Acquistition	Image Comparison
[25]	50.04	96.04	50		2^{98888}	Brush Coating, medium-cost, flex	UV scatteering	Pattern matching
[10]	-	-	-	-	-	Inkjet Printing, low-cost	UV fluorescence	AI assisted check
[26]	-	-	-	-	-	Laser Printing, Sputtering, high-cost	Light Propagation Interference	Image Comparison

Table S5.

* gianluca.fiori@unipi.it

[1] R. Sargeni, E. Dimaggio, F. Pieri, F. Fabbri, G. Pennelli, L. Colombo, G. Iannaccone, M. Macucci, and G. Fiori, "High-precision materials printer for fast prototyping of electronic devices based on 2d materials," *Advanced Materials Technologies*, vol. n/a, no. n/a, p. 2400610, 2024.

[2] C.-W. Chong, P. Raveendran, and R. Mukundan, "The scale invariants of pseudo-zernike moments," *Pattern Analysis & Applications*, vol. 6, no. 3, pp. 176–184, 2003.

[3] A. Maiti, J. Casarona, L. McHale, and P. Schaumont, "A large scale characterization of ro-puf," in *2010 IEEE International Symposium on Hardware-Oriented Security and Trust (HOST)*, pp. 94–99, 2010.

[4] A. Maiti, V. Gunreddy, and P. Schaumont, *A Systematic Method to Evaluate and Compare the Performance of Physical Unclonable Functions*. New York, NY: Springer New York, 2013.

[5] C. Gu, W. Liu, N. Hanley, R. Hesselbarth, and M. O'Neill, "A theoretical model to link uniqueness and min-entropy for puf evaluations," *IEEE Transactions on Computers*, vol. 68, pp. 287–293, 2 2019.

[6] N. Otsu, "A threshold selection method from gray-level histograms," *IEEE Transactions on Systems, Man, and Cybernetics*, vol. 9, no. 1, pp. 62–66, 1979.

[7] A. T. Erozan, M. Hefenbrock, M. Beigl, J. Aghassi-Hagmann, and M. B. Tahoori, "Image PUF: A physical unclonable function for printed electronics based on optical variation of printed inks," *Cryptology ePrint Archive: 2019/1419*, 2019.

[8] Y. Watanabe, K. Suemori, K. Kuribara, N. Fukuda, K. I. Nomura, and S. Uemura, "Development of a simple contact-type printable physically unclonable function device using percolation conduction of rod-like conductive fillers," *Japanese Journal of Applied Physics*, vol. 61, 6 2022.

[9] Y. Li, Z. Liu, K. Zhu, L. Ai, P. Jia, N. Wu, H. Yu, J. Wang, X. Yao, J. Zhou, and Y. Song, "Inkjet printed physically-unclonable structural-color anticounterfeiting labels with convenient artificial intelligence authentication," *Advanced Materials Interfaces*, vol. 8, 11 2021.

[10] Y. Gao, K. Ge, Z. Zhang, Z. Li, S. Hu, H. Ji, M. Li, and H. Feng, "Fine optimization of colloidal photonic crystal structural color for physically unclonable multiplex encryption and anti-counterfeiting," *Advanced Science*, vol. 11, 5 2024.

[11] Y. Liu, F. Han, F. Li, Y. Zhao, M. Chen, Z. Xu, X. Zheng, H. Hu, J. Yao, T. Guo, W. Lin, Y. Zheng, B. You, P. Liu, Y. Li, and L. Qian, "Inkjet-printed unclonable quantum dot fluorescent anti-counterfeiting labels with artificial intelligence authentication," *Nature Communications*, vol. 10, p. 2409, Jun 2019.

[12] H.-J. Jeon, J. W. Leem, Y. Ji, S. M. Park, J. Park, K.-Y. Kim, S.-W. Kim, and Y. L. Kim, "Cyber-physical watermarking with inkjet edible bioprinting," *Advanced Functional Materials*, vol. 32, no. 18, p. 2112479, 2022.

[13] A. F. Smith, P. Patton, and S. E. Skrabalak, "Plasmonic nanoparticles as a physically unclonable function for responsive anti-counterfeit nanofingerprints," *Advanced Functional Materials*, vol. 26, pp. 1315–1321, 3 2016.

[14] N. Sun, Z. Chen, Y. Wang, S. Wang, Y. Xie, and Q. Liu, "Random fractal-enabled physical unclonable functions with dynamic ai authentication," *Nature Communications*, vol. 14, 12 2023.

[15] M. S. Kim, M. H. Kang, J. S. Kim, Y. K. Hong, and G. J. Lee, "Flexible and wearable encryption primitive based on optical physically unclonable functions," *IEEE Journal of Selected Topics in Quantum Electronics*, vol. 30, pp. 1–8, 5 2024.

[16] Z. Man, C. Dong, J. Bian, Z. Lu, Y. Q. Lu, and W. Zhang, "Optically readable, physically unclonable subwavelength pixel via multicolor quantum dot printing for anticounterfeiting," *Nano Letters*, vol. 24, pp. 7019–7024, 6 2024.

[17] J. H. Kim, S. Jeon, J. H. In, S. Nam, H. M. Jin, K. H. Han, G. G. Yang, H. J. Choi, K. M. Kim, J. Shin, S. W. Son, S. J. Kwon, B. H. Kim, and S. O. Kim, "Nanoscale physical unclonable function labels based on block co-polymer self-assembly," *Nature Electronics*, vol. 5, pp. 433–442, 7 2022.

[18] D. I. Moon, A. Rukhin, R. P. Gandhiraman, B. Kim, S. Kim, M. L. Seol, K. J. Yoon, D. Lee, J. Koehne, J. W. Han, and M. Meyyappan, "Physically unclonable function by an all-printed carbon nanotube network," *ACS Applied Electronic Materials*, vol. 1, pp. 1162–1168, 7 2019.

[19] O. Read, K. Parvez, M. Boyes, X. Song, J. Wang, G. Fiori, and C. Casiraghi, "Novel anti-counterfeiting solution based on 2d materials produced by electrochemical exfoliation," *Small*, vol. 20, 4 2024.

[20] J. Zhang, R. Tan, Y. Liu, M. Albino, W. Zhang, M. M. Stevens, and F. F. Loeffler, "Printed smart devices for anti-counterfeiting allowing precise identification with household equip-

ment," *Nature Communications*, vol. 15, 12 2024.

[21] Y. W. Hu, T. P. Zhang, C. F. Wang, K. K. Liu, Y. Sun, L. Li, C. F. Lv, Y. C. Liang, F. H. Jiao, W. B. Zhao, L. Dong, and C. X. Shan, "Flexible and biocompatible physical unclonable function anti-counterfeiting label," *Advanced Functional Materials*, vol. 31, 8 2021.

[22] R. A. John, N. Shah, S. K. Vishwanath, S. E. Ng, B. Febriansyah, M. Jagadeeswararao, C. H. Chang, A. Basu, and N. Mathews, "Halide perovskite memristors as flexible and reconfigurable physical unclonable functions," *Nature Communications*, vol. 12, 12 2021.

[23] J. Zhang, Y. Liu, C. Njel, S. Ronneberger, N. V. Tarakina, and F. F. Loeffler, "An all-in-one nanoprinting approach for the synthesis of a nanofilm library for unclonable anti-counterfeiting applications," *Nature Nanotechnology*, vol. 18, pp. 1027–1035, 9 2023.

[24] Y. Li, Y. Li, Z. Zhao, Y. Li, F. Song, and W. Huang, "Multilevel stimuli–responsive smart "sandwich" label with physical unclonable functions bionic wrinkles and space-selective fluorescence patterns," *Small*, 12 2024.

[25] B. Lyu, Y. Ouyang, D. Gao, X. Wan, and X. Bao, "Multilevel and flexible physical unclonable functions for high-end leather products or packaging," *Small*, 2024.

[26] A. Ferraro, G. E. Lio, M. D. L. Bruno, S. Nocentini, M. P. D. Santo, D. S. Wiersma, F. Riboli, R. Caputo, and R. C. Barberi, "Hybrid camouflaged anticounterfeiting token in a paper substrate," *Advanced Materials Technologies*, vol. 8, 2 2023.
